# Supplementary material for: Region of Attainable Redaction, an extension of Ellipse of Insignificance analysis for gauging impacts of data redaction in dichotomous outcome trials
Source: eLife. 2024 Jan 29;13:e93050. doi: 10.7554/eLife.93050 (PMC10871715; doi:10.7554/eLife.93050)
Supplement: Supplementary file 1. [file elife-93050-supp1.pdf]

Region of Attainable Redaction, an extension of Ellipse of Insignificance analysis for gauging impacts of data redaction in dichotomous outcome trials - Mathematical Appendix

David Robert Grimes[1,2]

[1]School of Medicine, Trinity College Dublin, Dublin, Ireland
[2]School of Physical Sciences, Dublin City University, Dublin, Ireland
[*]davidrobert.grimes@dcu.ie / davidrobert.grimes@tcd.ie

ABSTRACT

Mathematical appendices for ROAR analysis - Form of $g(x,y)$ and $h(x,y)$.

Coefficients of $g(x,y)$

Table S1

Experimental arm with significantly higher risk ($RR_e > 1$)	
Variable	Coefficient
x^3y^2	1
x^3y	$2c$
x^3	c^2
x^2y^3	1
x^2y^2	$n+2(b+c)-\nu$
x^2y	$4bc+c^2-2ad+2cn-(a+2c+d)\nu$
x^2	$c(2bc-2ad+cn)-(a+c)(c+d)\nu$
xy^3	$2b$
xy^2	$b^2+4bc-2ad+2bn-(a+2b+d)\nu$
xy	$2(b+c)(bc-ad)+4bcn-2adn-(a+2b+d)(a+2c+d)\nu$
x	$(bc-ad)(bc-ad+2cn)-(a+c)(a+2b+d)(c+d)\nu$
y^3	b^2
y^2	$b(-2ad+b(2c+n))-(a+b)(b+d)\nu$
y	$(bc-ad)(-ad+b(c+2n))-(a+b)(b+d)(a+2c+d)\nu$
Constant term	$(bc-ad)^2n-(a+b)(a+c)(b+d)(c+d)\nu$
Experimental arm with significantly lower risk ($RR_e < 1$)	
Variable	Coefficient
x^3y^2	1
x^3y	$2d$
x^3	d^2
x^2y^3	1
x^2y^2	$n+2(a+d)-\nu$
x^2y	$4ad+d^2-2bc+2dn-(b+2d+c)\nu$
x^2	$d(2ad-2bc+dn)-(b+d)(c+d)\nu$
xy^3	$2a$
xy^2	$a^2+4ad-2bc+2an-(b+2a+c)\nu$
xy	$2(a+d)(ad-bc)+4adn-2bcn-(b+2a+c)(b+2d+c)\nu$
x	$(bc-ad)(bc-ad-2dn)-(b+d)(b+2a+c)(c+d)\nu$
y^3	a^2
y^2	$a(-2bc+a(2d+n))-(a+b)(a+c)\nu$
y	$(bc-ad)(bc-a(d+2n))-(a+b)(a+c)(b+2d+c)\nu$
Constant term	$(bc-ad)^2n-(a+b)(a+c)(b+d)(c+d)\nu$

Coefficients of resultant cubic curve $g(x,y)$

Coefficients of $h(x,y)$

Table S2

Experimental arm with significantly higher risk ($RR_e > 1$)	
Variable	Coefficient
x^4y	-2
x^4	$-2c$
x^3y^2	-3
x^3y	$-2(2b+2c+n-\nu)$
x^3	$-4bc-c^2+2ad-2cn+(a+2c+d)\nu$
x^2y^3	3
x^2y^2	$-6b+6c$
x^2y	$-2b^2+3c^2+4ad-4b(2c+n-\nu)+2(a+d)\nu$
x^2	$-2(b+c)(bc-ad)-4bcn+2adn+(a+2b+d)(a+2c+d)\nu$
xy^4	2
xy^3	$2(2b+2c+n-\nu)$
xy^2	$-3b^2+8bc+2c^2-4ad+4cn-2(a+2c+d)\nu$
xy	$2(b-c)(-2bc+2ad-bn-cn+n\nu)$
x	$-(bc-ad)(-ad+b(c+2n))+(a+b)(b+d)(a+2c+d)\nu$
y^4	$2b$
y^3	$b^2+4bc-2ad+2bn-(a+2b+d)\nu$
y^2	$2(b+c)(bc-ad)+4bcn-2adn-(a+2b+d)(a+2c+d)\nu$
y	$(bc-ad)(bc-ad+2cn)-(a+c)(a+2b+d)(c+d)\nu$
Experimental arm with significantly lower risk ($RR_e < 1$)	
Variable	Coefficient
x^4y	-2
x^4	$-2d$
x^3y^2	-3
x^3y	$-2(2a+2d+n-\nu)$
x^3	$-4ad-d^2+2bc-2dn+(b+2d+c)\nu$
x^2y^3	3
x^2y^2	$-6a+6d$
x^2y	$-2a^2+3d^2+4bc-4a(2d+n-\nu)+2(b+c)\nu$
x^2	$-2(a+d)(ad-bc)-4adn+2bcn+(b+2a+c)(b+2d+c)\nu$
xy^4	2
xy^3	$2(2a+2d+n-\nu)$
xy^2	$-3a^2+8ad+2d^2-4bc+4dn-2(b+2d+c)\nu$
xy	$2(a-d)(2bc-2ad-an-dn+n\nu)$
x	$-(bc-ad)(bc-a(d+2n))+(a+b)(a+c)(b+2d+c)\nu$
y^4	$2a$
y^3	$a^2+4ad-2bc+2an-(b+2a+c)\nu$
y^2	$2(a+d)(ad-bc)+4adn-2bcn-(b+2a+c)(b+2d+c)\nu$
y	$(bc-ad)(bc-ad-2dn)-(b+d)(b+2a+c)(c+d)\nu$

Coefficients of Lagrange derived polynomial $h(x,y)$
